# Cowhiding—More Blessed to Give Than to Receive

**Published:** 1890-03

**Authors:** 


### COWHIDING—MORE BLESSED TO GIVE THAN TO RECEIVE.

Cincinnati has had a sensation recently in a malpractice suit, which resulted in the attempt of two lawyers to cowhide the editor of a medical journal. In April, 1888, Dr. C. D. Palmer, Professor of Obstetrics and Diseases of Women and Clinical Gynecologist in the Medical College of Ohio, and Gynecologist to the Cincinnati Hospital, operated on a woman, and during the operation one of his needles broke and the piece could not be found. A few days later the doctor met with an accident, his horse running away, throwing him out under a culvert, whence he was taken up for dead. He was taken to the Good Samaritan Hospital, where he lay in a semi-unconscious state for weeks, and was unable to return to his professional work for a year. When able to travel he went to the Atlantic coast, and afterward to California, and regained his lost health and strength to such a degree that his practice, hospital and college work were resumed.

During this time, while he is many thousand miles away seeking health, the woman goes to another doctor, complaining of great pain. Another operation is performed and, behold, a piece of needle is brought forth. Recently Dr. Palmer received a letter from a youthful firm of briefless barristers, intimating that unless he came down with some money he would be sued for damages. He refused to consider their proposition, and was sued for $10,000. This action was followed by a scathing editorial in the next number of the *Lancet-Clinic*, January 25th, from its editor, Dr. J. C. Culbertson. His were words that burn, the actual cautery applied with unsparing hand, which cut to the bone, penetrating nerves and tissues in all directions. The limbs of the law read, writhed, condemned and sentenced the writer to a dose of cowhide. They sallied forth, with their cowhides fresh and new. They entered the doctor's office and, without presenting their cards, began using their pretty weapons. The editor, who was brought up on a big farm, soon recovered from his surprise, shook his powerful frame, took the weapons from the legal sprouts, gave them the dose they came to bestow upon him, walked to police headquarters with a cowhide in each hand and blood in his eye, and quieted official fears by telling them he had only come to swear out a warrant for assault and battery.

It is to be hoped that the action of these two attorneys will not only lead to the dismissal of the case with these two champions, but to the disbarment of the men who would resort to such means to retaliate on an editor. If lawyers cannot obtain redress at law, who can? The plain path for the medical profession to pursue is to stand together, to boycott lawyers who undertake these "bleeding" cases, destroy their business and political aspirations, where possible, and show that the medical profession is a unit, and a dangerous one to attack.—*Med. Rec.*

